# Risk of postpartum depression among women with endometriosis: the Norwegian mother, father and child cohort study (MoBa)

**DOI:** 10.1007/s10654-025-01338-2

**Published:** 2026-01-12

**Authors:** Marius Johansen, Tone Kristin Omsland, Katariina Laine, Siri Eldevik Håberg, Maria Christine Magnus

**Affiliations:** 1 Department of Community Medicine and Global Health, Institute of Health and Society, University of Oslo, Blindern, 1130, 0318 Oslo, Norway; 2 Norwegian Research Centre for Women’s Health, Oslo University Hospital, Oslo, Norway; 3 Faculty of Medicine, Institute of Clinical Medicine, University of Oslo, Oslo, Norway; 4 Centre for Fertility and Health, Norwegian Institute of Public Health, Oslo, Norway; 5 Department of Global Public Health and Primary Care, University of Bergen, Bergen, Norway

**Keywords:** Endometriosis, Postpartum depression, Infertility, Assisted reproductive technology, The Norwegian Mother, Father and Child Cohort Study, Medical Birth Registry of Norway

## Abstract

Women with endometriosis have a higher burden of anxiety and depression. Whether they are at increased risk of postpartum depression (PPD) remains unclear. We aimed to compare the risk of PPD between women with and without endometriosis and to explore mediation by previous history of major depression and infertility. In a population-based cohort study, we compared 1,159 singleton pregnancies to women with self-reported endometriosis and 74,590 pregnancies to women without endometriosis. We calculated a djusted risk ratios (aRR) with 95% confidence intervals (CI) using multivariable log-binomial regression, adjusting for age, body mass index, education and income. Mediation analyses assessed the indirect effect of any history of major depression or infertility. Women with endometriosis had a higher risk of PPD (aRR: 1.34, 95% CI: 1.15–1.55). Mediation analyses indicated that a large part of this association was explained by a higher lifetime prevalence of major depression among women with endometriosis (natural direct effect of endometriosis: aRR: 1.17, 95% CI: 1.00-1.36; natural indirect effect of any history of major depression: aRR: 1.14, 95% CI: 1.08–1.20), with 49.3% proportion mediated. Infertility demonstrated a negative natural indirect effect on the association between endometriosis and PPD (aRR: 0.87, 95% CI: 0.81–0.94). Women with endometriosis had an elevated risk of PPD which was largely explained by a higher lifetime prevalence of major depression. Our findings suggest that they constitute a high-risk group and could benefit from closer follow-up to facilitate early identification and intervention.

**Supplementary Information:**

The online version contains supplementary material available at 10.1007/s10654-025-01338-2.

## Introduction

Endometriosis is a prevalent chronic gynaecological condition, affecting around 10% of women of childbearing age, characterized by the growth of endometrial-resembling tissue outside the uterus, with pelvic pain, including dysmenorrhea, as the primary symptom [1, 2]. It may progress to chronic pain and dyspareunia [2, 3], significantly impairing quality of life [4, 5] and increasing the susceptibility to mental health disorders, including anxiety and depression [6, 7].

Postpartum depression (PPD) typically commences within the initial weeks postpartum or up to the first year of the child’s life [8], affecting approximately 17% of women globally [9]. PPD encompasses symptoms like anxiety, agitation, irritability, panic, anger, hypervigilance, and intrusive thoughts, including intense concerns about the ability to care for the newborn, heightened anxiety about the baby’s health, and severe feelings of parental inadequacy [8, 10, 11], which can interfere with daily functioning and the mother-child relationship [10]. These symptoms, accompanied by sadness, reduced energy, dysphoria, feelings of guilt or shame, difficulties in concentration, and suicidal ideation in severe cases, resemble those of general depression [8, 11]. This symptom overlap, along with substantial comorbidity with other perinatal mental disorders such as anxiety, mania, and psychosis, complicates PPD differentiation [8, 12]. Additionally, disorders present before pregnancy or recurring alongside those emerging during pregnancy or postpartum are considered perinatal mental illnesses [11], posing challenges in diagnosis and aetiology.

PPD risk factors are multifaceted, including genes, biological factors, hormonal changes, socio-environmental influences, psychological adjustment to motherhood, personal and family histories of depression and mental illness, and the stress associated with childbirth and caring for a new baby [11, 13, 14]. However, identifying exact causes is difficult because of the considerable differences in genetics, symptoms, and personal histories among women [8]. Endometriosis is associated with conditions impacting PPD risk, as women with endometriosis have a higher burden of major depression [6, 7], potentially predisposing them to develop PPD [13]. Conversely, their higher rates of infertility [15] may result in a lower risk of PPD, as evidenced by a recent systematic review indicating that women using artificial reproductive treatment (ART) had lower PPD incidence than those conceiving spontaneously (12 studies; combined OR: 0.83, 95% CI 0.71–0.97) [16]. Despite these associations, research examining the relationship between endometriosis and PPD remains scarce, with only one cohort study previously exploring this link [17]. This study from Japan showed an increased burden of endometriosis among women with PPD compared to those without the condition [17]. No studies have examined intermediate factors that might contribute to a higher burden of PPD among women with endometriosis.

Therefore, we aimed to compare the risk of PPD between women with and without endometriosis, and explore to what extent this association might be influenced by lifetime history of major depression or infertility.

## Materials and methods

### Study population

We studied women enrolled in the Norwegian Mother, Father, and Child Cohort Study (MoBa), a population-based pregnancy cohort initiated by the Norwegian Institute of Public Health. Between 1999 and 2008, approximately 95,000 pregnant women were recruited at around 18 gestational weeks across Norway, totalling approximately 112,000 pregnancies as some women participated with multiple pregnancies. Written informed consent was obtained, and the participation rate was 41% [18].

Self-reported information was collected through questionnaires during and after pregnancy and was supplemented with linkage to birth records from the Medical Birth Registry of Norway (MBRN) using unique identification numbers. The study protocol received approval from the Regional Committee for Medical and Health Research Ethics in South/East Norway (#2014/404).

### Endometriosis

Participants reported their health history at recruitment, including any diagnosis of endometriosis, without providing details on the diagnosis method or age at diagnosis. Those who indicated endometriosis before pregnancy in a yes/no format were categorized accordingly. We relied on self-reported diagnoses, which have been validated by previous research showing a relatively high agreement (72–95%) with medical records [19]. This validation supports the use of self-reported information as a viable method for identifying diagnosed endometriosis in epidemiological studies.

### Postpartum depression

The Edinburgh Postnatal Depression Scale (EPDS) is a 10-item questionnaire designed to capture symptoms typical of depression and anxiety in women during pregnancy and the first year postpartum [20]. In the MoBa study, participants completed a 4-item version of the EPDS at 6 months postpartum. This abbreviated version included the following items: “I have felt sad or miserable,” “I have been anxious or worried for no reason,” “I have been so unhappy that I have had difficulty sleeping,” and “I have blamed myself unnecessarily when things went wrong.” Responses were scored on a scale from 0 to 3, where 0 indicates “no, never,” 1 is “not very often,” 2 is “yes, now and then,” and 3 is “yes, almost all of the time.” The short 4-item version has been used in prior publications, with a cut-off score of ≥ 6 yielding a similar percentage of women with PPD as the full version of the scale does with a cut-off of ≥ 13 [21, 22].

### Covariates

Information on maternal age at delivery, parity, and use of ART were extracted from the MBRN. Registrations of ART included use of in vitro fertilization (IVF) with and without intracytoplasmic sperm injection (ICSI). From the MoBa-questionnaires, we further obtained self-reported data on lifetime history of major depression, pre-pregnancy weight and height (used to calculate body mass index [BMI; categorized as < 18.5, 18.5–24.9, 25-29.9, and >30]), educational attainment (categorized as less than high school, high school, up to 4 years of college, and >4 years of college), annual income (categorized as low [< 200,000 NOK (Norwegian krone)], medium [200,000-399,999 NOK], and high [>400,000 NOK]), and pregnancy planning at recruitment. Information on lifetime history of major depression was collected at recruitment using the Lifetime History of Major Depression scale, consisting of six questions [23]. The first five questions ask about specific symptoms lasting over two weeks: (1) feeling depressed or sad, (2) appetite issues, (3) lack of energy, (4) self-blame or worthlessness, and (5) concentration difficulties. The sixth question inquires whether three or more symptoms were experienced simultaneously. Each question is answered with a yes or no. Women who answered ‘Yes’ to both feeling depressed or sad and experiencing three or more of the symptoms simultaneously were defined as having lifetime history of major depression [23]. Women with planned pregnancies reported the time taken to conceive. Infertility was defined as the inability to conceive within 12 months or use of ART, encompassing both male and female infertility factors.

Maternal age, BMI, and socioeconomic status were identified as key confounders affecting both endometriosis (exposure) and PPD (outcome), while a lifetime history of major depression and infertility (including use of ART) were treated as intermediate factors [2, 9, 13, 24–28].

### Statistical analyses

We employed log-binomial regression for a complete case analysis to compare PPD risk between women with and without endometriosis. We calculated crude and adjusted relative risks (RR and aRR) with 95% confidence intervals (CI), adjusting standard errors for the dependency related to multiple pregnancies in the same women with robust cluster variance. Our multivariable model accounted for maternal age, BMI, and socioeconomic status (education and income). Stratified analyses explored PPD variations in women with endometriosis according to lifetime history of major depression and infertility. Interaction between exposure (endometriosis) and mediators (lifetime history of major depression and infertility) was tested by including product terms in the multivariable regression models. Mediation analyses quantified the total effect (TE), representing the causal association between endometriosis and PPD, the natural indirect effect (NIE), an estimate of the effect that goes through the selected mediators (lifetime history of major depression and infertility), and the natural direct effect (NDE), reflecting the effect of endometriosis on PPD not explained by the selected potential mediators [29]. The proportion mediated (PM) was calculated where both NDE and NIE deviated similarly from the null hypothesis, quantifying the percentage of the total effect that operates through the mediator. To further examine mediation via infertility, we conducted additional mediation analyses of (1) mediation through ART, regardless of time to pregnancy, and (2) mediation through prolonged infertility, defined as time to pregnancy exceeding 12 months without the use of ART. In this second analysis, women who conceived using ART were excluded. We also conducted sensitivity analyses restricted to nulliparous women. To evaluate the robustness of results obtained from the complete case analysis and address potential bias from missing data, we applied multiple imputation by chained equations to impute 20 datasets, including all 85,523 pregnancies from women who returned the MoBa questionnaires at recruitment and 6 months postpartum.

Stata version 18 (StataCorp, College station, TX, USA) was used for the analyses.

## Results

**Fig. 1:**
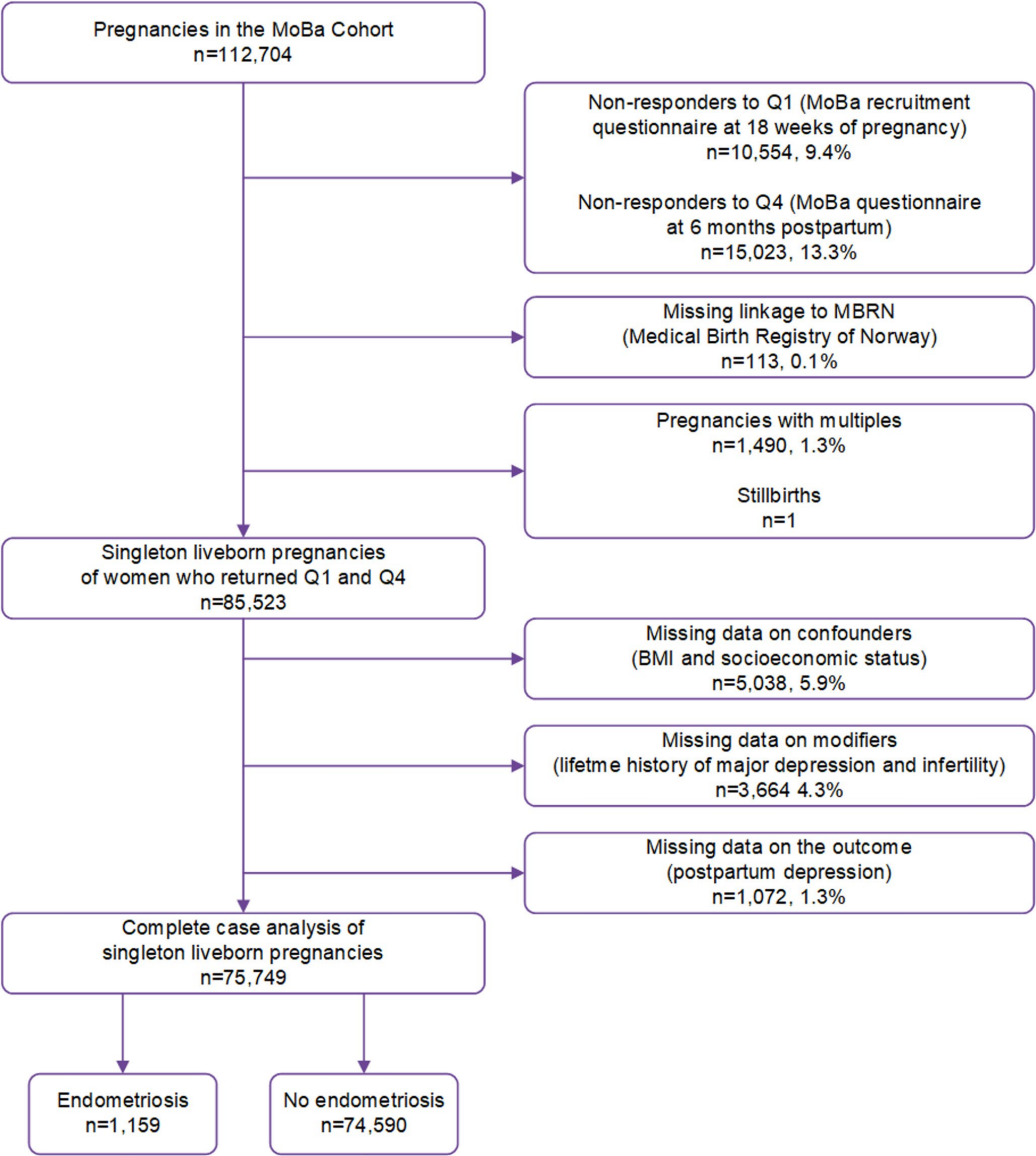
Illustration of study population. Flowchart displaying the inclusion of participants who returned the first (Q1) and the fourth questionnaires (Q4) of the Norwegian Mother, Father and Child Cohort Study (MoBa) with data from the Medical Birth Registry (MBRN), and exclusion of stillbirths, multiple pregnancies or those with missing information on one or more covariates

We restricted the analysis to liveborn singleton pregnancies to women with information from the questionnaires completed at 18 gestational weeks and 6 months postpartum. We excluded 9,887 pregnancies with missing linkage to MBRN or missing data on the outcome, confounders, or modifying factors, resulting in a study population of 75,749 pregnancies (Fig. 1). Endometriosis was self-reported by women in 1,159 pregnancies (1.5%), with the remaining 74,590 pregnancies classified as unexposed.

Women with endometriosis were generally older (32.9 vs. 30.7 years), more often nulliparous (50% vs. 46%), had lower BMI (8.0% with BMI > 30 vs. 9.3%), a higher income (15% with high annual income vs. 12%), were more likely to experience infertility (47% vs. 11%), and more likely to report a history of major depression (33% vs. 23%) (Table 1).

**Table 1:** Background characteristics according to endometriosis among 75,749 singleton pregnancies of women in the Norwegian Mother, Father and Child cohort study (1999-2008) based on a complete case analysis

	Endometriosis	No reported endometriosis
	n= 1,159 (1.5 %)	n= 74,590 (98.5 %)
Maternal age (SD)		
Age at birth, mean year	32.91 (4.09)	30.70 (4.47)
Parity		
Nulliparous	578 (49.87 %)	34,485 (46.23 %)
Multiparous	581 (50.13 %)	40,105 (53.77 %)
Body Mass Index		
<18.5	19 (1.64 %)	2,201 (2.95 %)
18.5-24.9	796 (68.68 %)	49,079 (65.80 %)
25-29.9	251 (21.66 %)	16,356 (21.93 %)
>30	93 (8.02 %)	6,954 (9.32 %)
Level of completed education		
Less than high school	79 (6.82 %)	4,755 (6.37 %)
High school	302 (26.06 %)	21,303 (28.56 %)
Up to 4 years of college	491 (42.36 %)	31,210 (41.84 %)
>4 years of college	287 (24.76 %)	17,322 (23.22 %)
Annual income		
Low (0-199.999 NOK)	232 (20.02 %)	21,026 (28.19 %)
Medium (200.000-399.999 NOK)	755 (65.14 %)	44,737 (59.98 %)
High (>400.000 NOK)	172 (14.84 %)	8,827 (11.83 %)
Lifetime history of major depression		
Yes	382 (32.96 %)	16,858 (22.60 %)
No	777 (67.04 %)	57,732 (77.40 %)
Mode of conception		
ART	259 (22.35 %)	1,444 (1.94 %)
No ART	900 (77.60 %)	73,146 (98.06 %)
Infertility^a^		
Yes	540 (46.59 %)	7,835 (10.50 %)
No	619 (53.41 %)	66,755 (89.50 %)

NOK: The Norwegian krone, currency of Norway, ART: Assisted reproductive technologies ^a^ Infertility: Failure to conceive within a year, using ART, or both

### Risk of postpartum depression

At six months postpartum, 10.3% had EPDS-scores indicating PPD. Women with endometriosis had a higher risk of PPD than those without the condition (13% vs. 10%), with an adjusted relative risk of 1.34 (95% CI: 1.15–1.55) (Table 2).

**Table 2:** Relative risk (RR) of postpartum depression among 75,749 singleton pregnancies in women with endometriosis in the Norwegian Mother, Father and Child cohort study (1999-2008) based on a complete case analysis (pregnancies with no reported endometriosis as reference group)

	Endometriosis	No reported endometriosis	Unadjusted RR	Adjusted RR^a^
	(n=1,159)	(n=74,590)		
Postpartum depression	154 (13.29 %)	7,679 (10.29 %)	1.29 (1.11-1.50)	1.34 (1.15-1.55)

^a^ Adjusted for maternal age at birth, BMI and socioeconomic status (maternal education and income)

The sensitivity analyses using multiple imputation for missing data on mediators, confounders, or outcomes across 85,523 pregnancies yielded similar results: the PPD risk among 1,293 women with endometriosis (aRR: 1.31, 95% CI: 1.14–1.51) was comparable to the complete case results (Online Resource 1).

### Stratified analyses and interaction tests

We observed that the risk of PPD among women with endometriosis varied depending on fertility status, with a significant interaction between endometriosis and infertility (interaction term p-value: 0.009). Women with endometriosis who conceived without fertility difficulties exhibited a higher PPD risk (17% vs. 10%; aRR: 1.61, 95% CI: 1.34–1.92), while women with infertility showed no increased risk (9.7% vs. 9.4%; aRR: 1.03, 95% CI: 0.78–1.34) (Online Resource 2). No significant differences were identified in the association between endometriosis and risk of PPD according to a history of major depression (interaction term p-value: 0.426) (Online Resource 2).

### Mediation analyses

We conducted two formal mediation analyses to evaluate the roles of lifetime history of major depression and infertility in the association between endometriosis and risk of PPD. Lifetime history of major depression showed a substantial mediating effect on the relationship between endometriosis and risk of PPD (NDE of endometriosis: aRR: 1.17, 95% CI: 1.00-1.36; and NIE through lifetime history of major depression: aRR: 1.14, 95% CI: 1.08–1.20), corresponding to a proportion mediated of 49.3%. In contrast, infertility exhibited an indirect effect below 1, consistent with risk reduction along the infertility pathway (NDE of endometriosis: aRR: 1.56, 95% CI: 1.31–1.84; and NIE through infertility: aRR: 0.87, 95% CI: 0.81–0.94) (Table 3). Secondary sub-analyses indicated similar indirect effects below 1 for ART use (NIE through ART: aRR: 0.94, 95% CI: 0.90–0.98) and for spontaneous conception after prolonged infertility (NIE through infertility with no use of ART: aRR: 0.97, 95% CI: 0.93–1.01) (Online Resource 3).

**Table 3:** Mediation analysis of the effect of endometriosis and mediators (lifetime history of major depression and infertility) on postpartum depression among 75,749 singleton pregnancies in the Norwegian Mother, Father and Child cohort study (1999-2008) based on a complete case analysis (relative risk for pregnancies in women with endometriosis, pregnancies with no reported endometriosis as reference group)^a^

Potential mediator	Endometriosis	Endometriosis	Endometriosis	Proportion mediated (PM) (%)
	Total effect (TE)	Natural direct effect (NDE)	Natural indirect effect (NIE)	
Lifetime history of major depression	1.33 (1.14-1.54)	1.17 (1.00-1.36)	1.14 (1.08-1.20)	49.3
Infertility	1.36 (1.17-1.58)	1.56 (1.31-1.84)	0.87 (0.81-0.94)	NA^b^

^a^ Adjusted for maternal age at birth, BMI and socioeconomic status (maternal education and income)

^b^ Infertility had a protective mediating effect on PPD risk, and as NDE and NIE deviated in opposite directions from the null hypothesis, PM could not be calculated

### Sensitivity analyses by parity

In the complete case cohort, 46% were nulliparous and 54% multiparous. Restricting our analyses to the 35,063 pregnancies of nulliparous women, we found similar differences in background characteristics when comparing nulliparous women with endometriosis (*n* = 578, 1.7%) to those without the condition (*n* = 34,485, 98.4%), except for a borderline difference in BMI (p-value: 0.069). Among nulliparous women, those with endometriosis had a slightly higher risk of PPD (aRR: 1.47, 95% CI: 1.20–1.81). Mediation analysis among nulliparous women showed the same patterns as in the entire cohort, with a substantial mediating effect of lifetime history of major depression on the relationship between endometriosis and risk of PPD, and a protective mediating effect of infertility on the PPD risk (Online Resource 4).

## Discussion

In this large Norwegian cohort, pregnant women with endometriosis exhibited a higher risk of PPD compared to those not reporting the condition. Mediation analyses indicated that a substantial proportion of this increased risk was explained by a higher prevalence of major depression among women with endometriosis. When examining the role of underlying infertility, it appeared that infertility resulted in a lower risk of PPD among women with endometriosis.

### Comparison with existing studies

Only one previous study has evaluated the association between PPD and endometriosis [17]. In this Japanese population-based cohort study of 82,489 participants, 14% experienced PPD at one month postpartum, which is slightly higher than our observed rate of 10.3%. The study reported an odds ratio (OR) of 1.27 (95% CI: 1.15–1.41), suggesting a higher risk of PPD among women with endometriosis, consistent with our findings.

Several differences exist between the Japanese study and ours. We used a shorter 4-item EPDS scale with a cut-off of 6 instead of their utilization of the full 10 item scale with a cut-off of 9. Additionally, we evaluated PPD at six months postpartum while they measured PPD at one month postpartum. Moreover, our adjustment strategy differed. The Japanese study adjusted for several factors that might be considered potential mediators, including for example parity, mode of conception, mode of delivery, and complications during pregnancy and birth, which may have caused them to underestimate the association.

Although cultural differences may influence the comparison of PPD prevalence across populations, a 2021 systematic review revealed only minor variations in the prevalence rates of PPD between Japan (27 studies; combined prevalence rate: 13.30%, 95% CI: 12.25–14.41) and Norway (12 studies; combined prevalence rate: 11.24%, 95% CI: 8.31–15.03) [9]. The systematic review identified national income and development as key predictors of PPD prevalence [9], and both Norway and Japan rank highly in these areas, making comparisons valid, and other cultural differences less significant in these comparisons.

Previous studies have not examined potential mediators, interaction effects, and the role of parity in the association between endometriosis and PPD.

### Potential explanatory mechanisms

Managing endometriosis often leads to fatigue, and pain catastrophizing [30], which may be compounded by the stress of surgical and medical treatments and their side effects [4]. Further, the condition has been found to aggravate mood alterations and depressive symptoms [6, 7], diminishing quality of life [4, 5]. Women affected by endometriosis frequently face stigmatization, trivialization, and inadequate support from partners, family, coworkers, and healthcare providers [5, 31]. This cumulative impact of symptoms and experiences leads to feelings of isolation, heightened stress reactions, and increases susceptibility to anxiety and depression [7, 32]. Furthermore, the hormonal state during pregnancy will often temporarily alleviate endometriosis symptoms due to menstrual cycle suppression, but emotional distress often arises when these symptoms reemerge postpartum [33].

The pathophysiology of endometriosis is characterized by proliferation of endometriotic tissue, followed by macrophage recruitment and cytokine secretion, which initiate localized inflammation that may progress to systemic inflammatory responses [3, 34]. Additionally, hormonal imbalances, including oestrogen dominance due to increased accumulation and synthesis of oestrogens and decreased sensitivity to progesterone, further drive proliferation and inflammation [2, 35]. With substantial inflammation, endometriosis shares features with autoimmune diseases, that may result in neurological effects by altering gene expression and volume in areas of the brain associated with pain sensitization and mood disorders, causing anxiety and depression [35].

Inflammatory pathways altering brain chemistry and stress responses, common to both endometriosis [34] and depression [28], may further link these conditions. Moreover, postpartum hormonal shifts, particularly oestrogen withdrawal, may increase PPD risk in women with a biological vulnerability, already susceptible to hormonal fluctuations [28].

We conducted mediation analysis to explore the relationship between endometriosis and PPD, aiming to determine whether intermediate factors could account for the observed effect. The analysis revealed that a substantial part of the increased PPD risk was mediated by a lifetime history of major depression, correlating with the higher prevalence of depression experiences earlier in life in women with endometriosis compared to those without endometriosis.

In contrast, infertility, or more precisely a successful pregnancy and live birth following infertility, was associated with a NIE below 1, consistent with reduced PPD risk along the infertility pathway. This suggests a potential protective effect of infertility-related factors. Our infertility subgroup analyses showed comparable protective mediating effects for both (1) pregnancies conceived using ART and (2) spontaneous conceptions following prolonged infertility, with the ART subgroup exhibiting a slightly stronger effect.

This potential protective effect may reflect the satisfaction and fulfilment of achieving a planned pregnancy after considerable effort [36], unlike the higher PPD risk observed after unintended pregnancies [37], which are more common among those who conceive without fertility difficulties. Additional contributing factors could include greater psychological preparedness, heightened desire for motherhood, or the “healthy survivor” effect, where highly motivated women with higher resilience, stronger psychological support and adequate financial resources are more likely to persist with treatment until conception [38]. Selection bias may also contribute to the observed effect, for example, if women undergoing ART are more likely to remain in the study while those experiencing PPD are more likely to drop out. Moreover, social desirability and reporting tendencies could contribute if some women feel pressure to report fewer depressive symptoms after overcoming infertility and receiving intensive support. Finally, enhanced clinical monitoring and access to supportive services in fertility care and ART programs may facilitate earlier identification and mitigation of perinatal mental health concerns.

While this study focused on examining two intermediary factors, other variables known to predict PPD risk were not accounted for, highlighting the need for future research to explore additional intermediary factors and potential pathways.

### Clinical implications

PPD can have serious short and long term consequences for both the mother [10], the child [39], and their dyadic interplay [40], posing substantial costs to society [41]. Globally, suicide is a leading cause of perinatal maternal death [42], underscoring the need for improved screening practices to address the often undetected and untreated cases of PPD [43]. Effective early detection through screening tools like the EPDS is cost-effective for preventing severe outcomes [44]. Simple screening interactions are inaccurate as a diagnostic tool, but are still valuable in identify at-risk women [45], ensuring timely intervention to prevent or lessen the negative effects associated with PPD. Considering endometriosis as a predictor of PPD could enhance detection and intervention strategies.

### Strengths and limitations

Strengths of this study include its novelty as one of the few investigations into the association between endometriosis and PPD. The large size and population-based sample of the MoBa-cohort are additional advantages. Moreover, it is the first study to consider mediation from lifetime history of major depression and infertility.

However, the study has limitations, such as utilizing a modified 4-item EPDS scale, which has also been employed in other recent publications [21, 22]. The absence of detailed information about the diagnosis method, stage, duration, and severity of endometriosis, along with reliance on unvalidated self-reported cases, presents challenges related to reporting and recall bias.

Nevertheless, we believe identifying endometriosis cases through self-reported diagnoses is valid, particularly in light of the substantial disparity observed in the use of ART (22% among those with endometriosis vs. 2% among those not reporting the condition). Our findings align with previous research showing significant correlations between endometriosis and associated conditions, such as a higher prevalence of lifetime history of major depression [7], ART reliance [46], and infertility [47]. However, the observed endometriosis prevalence rate of 1.5% is lower than the 2% reported in a 1997 Norwegian study [48], likely due to the underrepresentation of women with endometriosis-related infertility in a pregnancy-focused cohort. Our dataset, spanning 1999 to 2008, predates advances in understanding endometriosis, with current prevalence estimated at 10% [1]. Endometriosis is challenging to diagnose, with significant diagnostic delays probably leading to underdiagnosis [1, 2], which creates potential for underreporting and misclassification bias. If self-reported endometriosis misclassification is non-differential, the observed association with PPD could bias the true association, potentially masking a stronger relationship. Conversely, self-reporting likely captures women with severe symptoms, introducing selection bias that results in confounding, where severe symptoms lead to both diagnosis and PPD, inflating the observed association. These considerations necessitate caution when relating our findings to current practices.

The timeframe for defining PPD is debated, with some experts favouring a stricter definition of 4 weeks to 3 months postpartum [11]. Administering the EPDS earlier would enable a more valid comparison with the Japanese cohort study, which assessed PPD at 1-month postpartum [17]. However, in our study, the MoBa questionnaire was first administered six months postpartum, with no earlier postpartum data available due to the nature of data collection. Assessing EPDS earlier could also mitigate limitations in data interpretation caused by uncertainties about whether women received PPD detection or follow-up, like psychotherapy or antidepressants. Additionally, it would help address gaps related to factors potentially influencing depressive episode remission, such as changes in sleep schedules, return to work, and additional childcare support, and which may explain the lower PPD rates compared to the Japanese study [17]. While we anticipate these interventions to be evenly distributed among groups, individuals with endometriosis may have had more healthcare follow-up due to ART involvement, potentially leading to an underestimated prevalence of PPD in this group.

A 41% participation rate in the MoBa-cohort, skewed toward higher socioeconomic backgrounds [49], raises questions about generalizability to the broader endometriosis population. However, selection into MoBa does not inherently bias PPD estimates [50], as the primary objective was to examine associations. Although limited to the MoBa cohort, the comprehensive data and health registry linkage provide robust insights into endometriosis and PPD in Norway. We urge future studies, including newer cohorts from other countries, to broaden these findings, enhance generalizability, and explore causal mechanisms.

## Conclusions

Women with endometriosis have an elevated risk of PPD, partly explained by a higher burden of a history of major depression. While further studies are needed to replicate these findings, the results suggest that women with endometriosis constitute a high-risk group for PPD, and that they might benefit from closer follow-up to initiate early interventions.

## Supplementary Information

Below is the link to the electronic supplementary material.

Online Resource 1: Assessment of potential bias from missing data: Tables of background characteristics and risk of PPD, including those with missing values on outcome, confounders, and modifying factors. Supplementary Material 1

Online Resource 2: Complete tables of stratified and interaction analyses. Supplementary Material 2

Online Resource 3: Mediation of the association between endometriosis and PPD via ART and prolonged infertility (>12 months without ART). Supplementary Material 3

Online Resource 4: Table of sensitivity analyses related to nulliparity. Supplementary Material 4
