# Supplementary material for: Risk of postpartum depression among women with endometriosis: the Norwegian mother, father and child cohort study (MoBa)
Source: Eur J Epidemiol. 2026 Jan 12;41(2):197–206. doi: 10.1007/s10654-025-01338-2 (PMC12975844; doi:10.1007/s10654-025-01338-2)
Supplement: Supplementary file 1 — Online Resource 1: Assessment of potential bias from missing data: Tables of background characteristics and risk of PPD, including those with missing values on outcome, confounders, and modifying factors. Supplementary Material 1 [file 10654_2025_1338_MOESM1_ESM.pdf]

Supplemental Table 1a. Background characteristics according to endometriosis for singleton women among 85,523 pregnancies in the Norwegian Mother, Father and Child cohort study (1999-2008) with missing values on outcome, confounders, and modifying factors.

	Endometriosis n=1,293 (1.5 %)		No reported endometriosis n=84,230 (98.5 %)	
**Maternal age (SD)**				
Age at birth, mean year	32.94	(4.09)	30.1	(4.51)
Missing	*0*		*0*	
**Parity**				
Nulliparous	639	(49.42 %)	38,347	(45.53 %)
Multiparous	654	(50.58 %)	45,883	(54.47 %)
Missing	*0*		*0*	
**Body Mass Index**				
<18.5	26	(2.06 %)	2,459	(2.99 %)
18.5-24.9	870	(68.88 %)	54,100	(65.89 %)
25-29.9	267	(21.14 %)	17,953	(21.86 %)
>30	100	(7.92 %)	7,600	(9.26 %)
Missing	*30*		*2,148*	
**Level of completed education**				
Less than high school	91	(7.08 %)	5,865	(6.99 %)
High school	344	(26.77 %)	24,328	(29.01 %)
Up to 4 years of college	529	(41.17 %)	34,530	(41.17 %)
>4 years of college	321	(24.98 %)	19,150	(22.83 %)
Missing	*8*		*357*	
**Annual income**				
Low (0-199.999 NOK)	254	(20.16 %)	23,425	(28.74 %)
Medium (200.000-399.999 NOK)	816	(64.76 %)	48,606	(59.63 %)
High (>400.000 NOK)	190	(15.08 %)	9,480	(11.63 %)
Missing	*33*		*2,719*	
**Lifetime history of major depression**				
Yes	414	(32.88 %)	18,588	(22.63 %)
No	845	(67.12 %)	63,545	(77.37 %)
Missing	*34*		*2,097*	
**Mode of conception**				
ART	285	(22.04 %)	1,561	(1.85 %)
No ART	1,008	(77.96 %)	82,669	(98.15 %)
Missing	*0*		*0*	
**Infertility**[a]				
Yes	586	(46.47 %)	8,533	(10.44 %)
No	675	(53.53 %)	73,199	(89.56 %)
Missing	*32*		*2,498*	
**PPD**				
Yes	170	(13.31 %)	8,738	(10.54 %)
No	1,107	(86.69 %)	74,153	(89.46 %)
Missing	*16*		*1,339*	

Abbreviations: EPDS: Edinburgh postpartum depression scale, NOK: the Norwegian krone, currency of Norway, ART: assisted reproductive technologies, PPD: postpartum depression

[a]*Infertility: failure to conceive within a year, using ART, or both*

Supplemental Table 1b. Relative risk (RR) of postpartum depression for singleton pregnancies of women with endometriosis among 85,523 pregnancies in MoBa calculated with multiple imputation, with missing values on outcome, confounders, and modifying factors (pregnancies with no reported endometriosis as reference group).

	Endometriosis (n=1,293)	No endometriosis (n=84,230)	Unadjusted RR	Adjusted RR[a]
PPD	170 (13.31 %)	8,738 (10.54 %)	1.26 (1.09-1.45)	1.31 (1.14-1.51)

[a] adjusted for maternal age at birth, BMI and socioeconomically status (maternal education and income)

Abbreviations: PPD: postpartum depression

Article title:

Risk of postpartum depression among women with endometriosis: the Norwegian Mother, Father and Child Cohort Study (MoBa)

Journal name:

European Journal of Epidemiology

Author names:

Marius Johansen MD, Tone Kristin Omsland PhD, Katariina Laine PhD, Siri Eldevik Håberg PhD, Maria Christine Magnus PhD

Corresponding Author:

Marius Johansen

Institute of Health and Society, University of Oslo

P.O. Box 1130 Blindern, 0318 Oslo

Email: mariuj@medisin.uio.no
