# Supplementary material for: Risk of postpartum depression among women with endometriosis: the Norwegian mother, father and child cohort study (MoBa)
Source: Eur J Epidemiol. 2026 Jan 12;41(2):197–206. doi: 10.1007/s10654-025-01338-2 (PMC12975844; doi:10.1007/s10654-025-01338-2)
Supplement: Supplementary file 2 — Online Resource 2: Complete tables of stratified and interaction analyses. Supplementary Material 2 [file 10654_2025_1338_MOESM2_ESM.pdf]

Supplemental Table 2a. Relative risk (RR) of postpartum depression in women with endometriosis stratified by *lifetime history of major depression* (pregnancies with no reported endometriosis as reference), with interaction terms (p-value) assessing the interaction between endometriosis and having had a lifetime history of major depression, among 75,749 singleton pregnancies in the Norwegian Mother, Father and Child cohort study (1999-2008).

	**Lifetime history of major depression**						**No lifetime history of major depression**						**Interaction**
	No endometriosis (n=16,858)		Endometriosis (n=382)				No endometriosis (n=57,732)		Endometriosis (n=777)				
	n	%	n	%	Unadjusted RR	Adjusted RR[a]	n	%	n	%	Unadjusted RR	Adjusted RR[a]	p-value
Postpartum depression	3,680	21.83	99	25.92	1.19 (1.00-1.41)	1.24 (1.04-1.47)	3,999	6.93	55	7.08	1.02 (0.79-1.32)	1.07 (0.83-1.40)	0.426

[a]*Adjusted RR: adjusted for maternal age at birth, BMI and socioeconomic status (maternal education and income)*

Supplemental Table 2b. Relative risk (RR) of postpartum depression in women with endometriosis stratified by *fertility* (pregnancies with no reported endometriosis as reference), with interaction terms (p-value) assessing the interaction between endometriosis and being categorized as infertile, among 75,749 singleton pregnancies in the Norwegian Mother, Father and Child cohort study (1999-2008).

	**Infertile**						**Fertile**						**Interaction**
	No endometriosis (n=7,835)		Endometriosis (n=540)				No endometriosis (n=66,755)		Endometriosis (n=619)				
	n	%	n	%	Unadjusted RR	Adjusted RR[a]	n	%	n	%	Unadjusted RR	Adjusted RR[a]	p-value
Postpartum depression	763	9.74	51	9.44	0.97 (0.74-1.27)	1.03 (0.78-1.34)	6,916	10.36	103	16.64	1.61 (1.34-1.92)	1.61 (1.34-1.92)	0.009[*]

[a]*Adjusted RR: adjusted for maternal age at birth, BMI and socioeconomic status (maternal education and income)*

[*]*Statistically significant interaction term ($p<0.05$) (assessing the interaction between endometriosis and infertility)*

Article title:

Risk of postpartum depression among women with endometriosis: the Norwegian Mother, Father and Child Cohort Study (MoBa)

Journal name:

European Journal of Epidemiology

Author names:

Marius Johansen MD, Tone Kristin Omsland PhD, Katariina Laine PhD, Siri Eldevik Håberg PhD, Maria Christine Magnus PhD

Corresponding Author:

Marius Johansen

Institute of Health and Society, University of Oslo

P.O. Box 1130 Blindern, 0318 Oslo

Email: mariuj@medisin.uio.no
