# Supplementary material for: Risk of postpartum depression among women with endometriosis: the Norwegian mother, father and child cohort study (MoBa)
Source: Eur J Epidemiol. 2026 Jan 12;41(2):197–206. doi: 10.1007/s10654-025-01338-2 (PMC12975844; doi:10.1007/s10654-025-01338-2)
Supplement: Supplementary file 3 — Online Resource 3: Mediation of the association between endometriosis and PPD via ART and prolonged infertility (>12 months without ART). Supplementary Material 3 [file 10654_2025_1338_MOESM3_ESM.pdf]

Supplemental Table 3. Mediation analysis of the effect of endometriosis and two mediators: (1) use of assisted reproductive technology (ART) and (2) spontaneous conception after prolonged infertility (time to pregnancy >12 months without ART) on the risk of postpartum depression among 75,749 singleton pregnancies in the Norwegian Mother, Father and Child cohort study (1999-2008) based on a complete case analysis (relative risk for pregnancies in women with endometriosis, pregnancies with no reported endometriosis as reference group)[a].

Potential mediator	Endometriosis<br>Total effect (TE)	Endometriosis<br>Natural direct effect (NDE)	Endometriosis<br>Natural indirect effect (NIE)	Proportion mediated(PM) (%)
ART	1.36 (1.18-1.69)	1.45 (1.24-1.69)	0.94 (0.90-0.98)	NA[b]
Spontaneous conception after prolonged infertility	1.35 (1.16-1.56)	1.39 (1.19-1.63)	0.97 (0.93-1.01)	NA[b]

Abbreviations: ART: assisted reproductive technologies

[a] *adjusted for maternal age at birth, BMI and socioeconomic status (maternal education and income)*

[b] *the mediators had a protective mediating effect on PPD risk, and as NDE and NIE deviated in opposite directions from the null hypothesis, PM could not be calculated*

Article title:

Risk of postpartum depression among women with endometriosis: the Norwegian Mother, Father and Child Cohort Study (MoBa)

Journal name:

European Journal of Epidemiology

Author names:

Marius Johansen MD, Tone Kristin Omsland PhD, Katariina Laine PhD, Siri Eldevik Håberg PhD, Maria Christine Magnus PhD

Corresponding Author:

Marius Johansen

Institute of Health and Society, University of Oslo

P.O. Box 1130 Blindern, 0318 Oslo

Email: mariuj@medisin.uio.no
