# Supplementary material for: Risk of postpartum depression among women with endometriosis: the Norwegian mother, father and child cohort study (MoBa)
Source: Eur J Epidemiol. 2026 Jan 12;41(2):197–206. doi: 10.1007/s10654-025-01338-2 (PMC12975844; doi:10.1007/s10654-025-01338-2)
Supplement: Supplementary file 4 — Online Resource 4: Table of sensitivity analyses related to nulliparity. Supplementary Material 4 [file 10654_2025_1338_MOESM4_ESM.pdf]

Supplemental Table 4a. Background characteristics according to endometriosis among 35,063 singleton pregnancies in nulliparous women in the Norwegian Mother, Father and Child cohort study (1999-2008) based on a complete case analysis.

	Endometriosis n=578 (1.7 %)		No reported endometriosis n=34,485 (98.4 %)	
**Maternal age (SD)**				
Age at birth, mean year	31.92	(4.10)	28.97	(4.30)
**Body Mass Index**				
<18.5	9	(1.56 %)	1,157	(3.36 %)
18.5-24.9	414	(71.63 %)	23,499	(68,14 %)
25-29.9	110	(19.03 %)	6,953	(20.16 %)
>30	45	(7.79 %)	2,876	(8.34 %)
**Level of completed education**				
Less than high school	32	(5.54 %)	1,887	(5.47 %)
High school	145	(25.09 %)	9,859	(28.59 %)
Up to 4 years of college	251	(43.43 %)	14,419	(41.81 %)
>4 years of college	150	(25.95 %)	8,320	(24.13 %)
**Annual income**				
Low (0-199.999 NOK)	91	(15.74 %)	9,245	(26.81 %)
Medium (200.000-399.999 NOK)	387	(66.96 %)	20,966	(60.80 %)
High (>400.000 NOK)	100	(17.30 %)	4,274	(12.39 %)
**Lifetime history of major depression**				
Yes	199	(34.43 %)	7,713	(22.37 %)
No	379	(65.57 %)	26,772	(77.63 %)
**Mode of conception**				
ART	184	(31.83 %)	1,010	(2.93 %)
No ART	394	(68.17 %)	33,475	(97.07 %)
**Infertility**[a]				
Yes	355	(61.42 %)	4,560	(13.22 %)
No	223	(38.58 %)	29,925	(86.78 %)

Abbreviations: NOK: the Norwegian krone, currency of Norway, ART: assisted reproductive technologies

[a]*Infertility: failure to conceive within a year, using ART, or both*

Supplemental Table 4b. Relative risk (RR) of postpartum depression among 35,063 singleton pregnancies in nulliparous women with endometriosis in the Norwegian Mother, Father and Child cohort study (1999-2008) based on a complete case analysis (pregnancies with no reported endometriosis as reference group).

	Endometriosis (n=578)	No reported endometriosis (n=34,485)	Unadjusted RR	Adjusted RR[a]
Postpartum depression	80 (13.84 %)	3,517 (10.20 %)	1.35 (1.10-1.67)	1.47 (1.20-1.81)

[a] *adjusted for maternal age at birth, BMI and socioeconomic status (maternal education and income)*

Supplemental Table 4c. Mediation analysis of the effect of endometriosis and mediators (lifetime history of major depression and infertility) on postpartum depression among 35,063 singleton pregnancies in nulliparous women in the Norwegian Mother, Father and Child cohort study (1999-2008) based on a complete case analysis (relative risk for pregnancies in individuals with endometriosis, pregnancies with no reported endometriosis as reference group)[a].

Potential mediator	Endometriosis Total effect (TE)	Endometriosis Natural direct effect (NDE)	Endometriosis Natural indirect effect (NIE)	Proportion mediated (PM) (%)
Lifetime history of major depression	1.44 (1.18-1.76)	1.28 (1.04-1.58)	1.12 (1.05-1.20)	36.4
Infertility	1.53 (1.25-1.87)	1.95 (1.54-2.47)	0.78 (0.70-0.88)	b

[a] *adjusted for maternal age at birth, BMI and socioeconomic status (maternal education and income)*

[b] *infertility had a protective mediating effect on PPD risk, and as NDE and NIE deviated in opposite directions from the null hypothesis, PM could not be calculated*

Article title:

Risk of postpartum depression among women with endometriosis: the Norwegian Mother, Father and Child Cohort Study (MoBa)

Journal name:

European Journal of Epidemiology

Author names:

Marius Johansen MD, Tone Kristin Omsland PhD, Katariina Laine PhD, Siri Eldevik Håberg PhD, Maria Christine Magnus PhD

Corresponding Author:

Marius Johansen

Institute of Health and Society, University of Oslo

P.O. Box 1130 Blindern, 0318 Oslo

Email: mariuj@medisin.uio.no
